# Comparative cone beam computed tomography analysis of root canal morphology in permanent maxillary and mandibular canines within a Saudi Arabian subpopulation in the Albaha region

**DOI:** 10.25122/jml-2026-0032

**Published:** 2026-06

**Authors:** Mohammed Sarhan Alzahrani

**Affiliations:** 1 Restorative Dental Sciences Department, College of Dentistry, Al-Baha University, Al-Baha, Saudi Arabia

**Keywords:** cone-beam computed tomography, dental pulp cavity, anatomy, cross-sectional, cuspid, Saudi Arabia

## Abstract

The successful outcome of endodontic therapy is intrinsically linked to a comprehensive understanding of root canal anatomy and its frequent variations. The objective of this study was to evaluate the root and root canal morphology of permanent maxillary and mandibular canines in a Saudi subpopulation from the Al-Baha region using cone-beam computed tomography (CBCT), specifically investigating the prevalence of Vertucci classifications and canal shape variations across different root levels. This retrospective study analyzed 120 CBCT scans of permanent canines. Internal anatomy was categorized according to Vertucci’s classification, and cross-sectional canal shapes were assessed at the cervical, middle, and apical thirds using a standardized 10-point scale. Data were analyzed using Chi-square tests and multivariate logistic regression to assess statistical significance (*P* < 0.05) by gender and dental arch. All examined teeth were single-rooted. While Vertucci Class I was the most prevalent configuration overall, mandibular canines exhibited significantly greater morphological complexity in the middle third than maxillary canines (*P* = 0.016), including an 8.5% prevalence of Class III configurations. Morphologically, the most frequent transition in canal shape was from oval at the cervical level to circular at the apical third. This CBCT-based analysis demonstrates that, although single canals predominate, mandibular canines in this subpopulation exhibit significant internal complexity and anatomical variation. These findings underscore the necessity for clinicians to utilize advanced imaging and adapt disinfection protocols to address the specific morphological transitions—such as oval-to-circular shapes and Class III configurations—identified in the mandibular arch to improve endodontic outcomes.

## Introduction

The complexity of root canal anatomy poses significant challenges in endodontics, making it imperative that clinicians possess detailed knowledge of these variations. Therefore, understanding of root canal anatomy and its frequent variations is a crucial key for successful root canal treatment [1]. The primary objectives of root canal therapy—debridement and disinfection—cannot be achieved if the clinician fails to identify and treat all existing canals within a tooth [2]. Among the permanent dentition, canines are often perceived as having simple, single-canal systems; however, contemporary research highlights significant morphological complexity that can challenge standard clinical expectations [3-5]. While maxillary canines are remarkably stable, mandibular canines frequently exhibit complex internal architectures, including bifurcations and multiple apical foramina.

The internal configuration of root canals is most commonly categorized using the Vertucci classification system [6]. This system identifies eight distinct types of canal patterns, ranging from simple single canals (Type I) to complex configurations where canals split and rejoin (Type III) or remain separate (Type V). Understanding the prevalence of these classes is critical, as failing to recognize a complex canal configuration often leads to the creation of untreated canal space and subsequent endodontic failure.

Beyond the number of canals, the cross-sectional shape of the canal at different levels—cervical, middle, and apical—plays a vital role in the biomechanical preparation of the root canals. The canal shape determines the selection of rotary files and the efficiency of irrigation [7].

The advent of cone-beam computed tomography (CBCT) has fundamentally transformed the assessment of endodontic anatomy by overcoming the inherent limitations of traditional radiography [8,9]. Conventional periapical radiographs often fail to capture the complexity of the root canal system due to anatomical superimposition and the compression of three-dimensional structures into two-dimensional images [3,8]. In contrast, CBCT provides high-resolution, non-invasive, three-dimensional visualization, enabling clinicians to accurately identify intricate canal configurations, detect supplemental or missed canals, and analyze axial cross-sections at multiple root levels [2,10]. These detailed insights into internal architecture are essential for effective mechanical preparation and disinfection, significantly reducing the risk of procedural failure [1,7].

In the Saudi Arabian population, anatomical diversity in the permanent dentition has been widely documented. Yet, localized studies in regions such as Al-Baha are essential for understanding specific subpopulation trends [11]. The diversity of root canal morphology is not only a matter of individual variation but is also significantly influenced by ethnic and racial backgrounds. The literature has consistently shown that the prevalence of specific canal configurations, such as multiple canals in mandibular anterior teeth or C-shaped systems, varies across populations [12]. For instance, certain anatomical traits may be more frequent in Asian or African populations than in Caucasian populations, suggesting a strong genetic component in the development of the internal dental architecture. Therefore, population-specific studies are essential to provide clinicians with geographically relevant data, as relying on global averages may lead to a misunderstanding of the local population’s unique endodontic challenges [11,12].

The objective of this investigation was to provide a comprehensive analysis of the root canal morphology of permanent canines in a Saudi subpopulation within the Al-Baha region. By evaluating the number of roots, canal configurations according to Vertucci, and cross-sectional shapes at three distinct levels, this study aims to correlate these findings with gender and dental arch. Ultimately, these results will serve as a clinical guide to improve the precision of endodontic diagnosis and treatment for patients in this region. The null hypothesis was that there would be no significant differences in the root canal morphology and cross-sectional shapes between permanent maxillary and mandibular canines within the studied Saudi Arabian subpopulation.

## Material and methods

### Study design

The methodology for this retrospective study was thoughtfully designed to evaluate the internal morphology of permanent canines. We drew upon a digital database composed of CBCT images from the Saudi subpopulation in the Al-Baha region. This careful approach ensures that the findings accurately reflect the anatomical variations pertinent to effective endodontic treatment. The assessment of each tooth involved a multi-planar reconstruction (MPR) analysis to record the number of roots and the internal canal configuration. The canal types were categorized according to the Vertucci classification system (Classes I through VIII). To ensure accuracy, each scan was examined from the pulp chamber to the apex in axial, sagittal, and coronal views, enabling identification of splits, merges, and accessory canals.

The internal canal shape was analyzed at three distinct horizontal levels: the cervical third, the middle third, and the apical third. Using a standardized 10-point scale, shapes were classified according to the Bueno classification as circular, conical-pyramidal, oval/long oval, flattened/ribbon, eight-shaped, c-shape, calcified, trapezoidal, drop-shaped, or others [10]. This granular analysis provided a detailed map of the canal’s volumetric transition as it tapered toward the root tip.

### Sample selection

A random sample of 120 CBCT scans, comprising 60 maxillary and 60 mandibular permanent canines, was analyzed using iCAT® software. The study was conducted in the Al-Baha region, the smallest region in Saudi Arabia, with a population density more than five times lower than that of other major regions. Due to this smaller population size, the selected sample provides sufficient statistical power to represent the local demographic. The sample size calculation was further validated using G*Power software to ensure a 95% confidence level. A post-hoc power analysis confirmed that this sample size was robust; with an observed large effect size (adjusted odds ratio (aOR) = 5.42) and alpha = 0.05), the study achieved a statistical power exceeding 99%, ensuring that the findings regarding arch-based morphological variations are statistically reliable. These wide-field-of-view scans (6x8) were acquired retrospectively for various diagnostic purposes using a Gendex GXDP-700 Panoramic CBCT X-ray device (KaVo Kerr Group, USA) with the following parameters: 90 kVp, 12.5 mA, 5-second exposure time, and a 300 μm voxel size. Inclusion criteria included teeth with fully developed apices, no history of endodontic treatment, and high-quality, artifact-free radiographs.

### Data analysis

A random sample of 20 CBCT scans was re-examined by the same observer 5 days after the initial evaluation. Intra-examiner agreement for Vertucci classification and cross-sectional shape was calculated using Cohen’s Kappa coefficient, yielding a value of [0.94], representing almost perfect agreement.

Data on the subject’s gender and the specific tooth position (Universal Numbering System) were recorded to enable comparative analysis. All data points—including the number of canals at each level and the corresponding shape—were entered into a digital spreadsheet for subsequent statistical processing.

The collected data were processed and analyzed using IBM SPSS Statistics (Version 25.0) and Microsoft Excel to ensure high precision in frequency and correlation calculations. Statistical analysis was conducted to determine the frequency and distribution of each morphological trait. Fisher’s Exact test was utilized to assess significant differences between genders and between maxillary and mandibular arches, with a significance threshold set at *P* < 0.05. To address potential confounding factors and explore the interaction between variables, a multivariate logistic regression analysis was performed. The dependent variable was ‘canal complexity’ (categorized as simple [Class I] vs. complex [Class II–VI]). Independent variables included arch (maxillary vs. mandibular) and gender (male vs. female). Odds ratios (ORs) and 95% confidence intervals (CIs) were calculated to assess the strength of these associations. *P* values < 0.05 were considered statistically significant. This rigorous approach ensured that the variations observed were representative of the unique dental characteristics of the Al-Baha subpopulation.

## Results

A total of 120 permanent canines, including 60 maxillary and 60 mandibular canines, were selected and investigated from a digital database of a Saudi subpopulation in the Al-Baha region. In this specific dataset, 99.16% of permanent canines were single-rooted (*n* = 119), regardless of internal canal complexity. Only one mandibular canine presented with two roots. Mandibular canines exhibit significantly higher morphological complexity, with 25% showing multiple canals at the middle or apical levels compared to less than 5% in maxillary canines (Table 1).

**Table 1:** The frequency of Vertucci’s classification of the root canals of the maxillary and mandibular canines

Vertucci Class	Maxillary Canines	Mandibular Canines	*P* value
**Class I (1-1)**	56 (93.3%)	43 (71.7%)	0.016*
**Class II (2-1)**	0 (0%)	4 (6.7%)	
**Class III (1-2-1)**	3 (5.0%)	9 (15.0%)	
**Class V (1-2)**	1 (1.7%)	3 (5.0%)	
**Other (Type VI/Unclear)**	0 (0%)	1 (1.6%)	

There was a statistically significant difference in the distribution of Vertucci root canal configurations between maxillary and mandibular canines (*P* = 0.030). Specifically, mandibular canines showed a significantly higher prevalence of complex canal types (Classes II, III, and V) compared to maxillary canines, which are predominantly Class I (93.3%). There was no statistically significant difference in root canal complexity between males and females in this study (*P* = 0.473; Table 2).

**Table 2:** Comparing the complexity (Vertucci Class > I) between men and women

Gender	Total Canines	Complex Morphology (Class II+)	% Complexity	*P* value
**Male**	62	9	14.5%	0.473
**Female**	58	12	20.7%	

**Figure 1:**
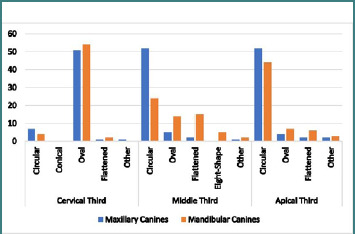
Frequency distribution of root canal cross-sectional morphologies in maxillary and mandibular permanent canines across 3 root thirds. Grouped bar chart comparing the prevalence of canal shapes (circular, conical, oval, flattened, eight-shape, and other) at the cervical, middle, and apical thirds for maxillary canines (blue) and mandibular canines (orange).

The most common morphological cross-sectional shape for canines in this dataset was oval in the cervical third and circular in the middle and apical third, as shown in Table 3 and Figure 1.

**Table 3:** The frequency of specific canal shapes at three different levels of the root, comparing maxillary and mandibular teeth

Root Canal Level	Canal Shape	Maxillary Canines	Mandibular Canines	Total	*P* value
**Cervical Third**	Circular	7	4	11	0.678
	Oval	51	54	105	
	Flattened	1	2	3	
	Other	1	0	1	
**Middle Third**	Circular	52	24	76	0.0054*
	Oval	5	14	19	
	Flattened	2	15	17	
	Eight-Shape	0	5	5	
	Other	1	2	3	
**Apical Third**	Circular	52	44	96	0.655
	Oval	4	7	11	
	Flattened	2	6	8	
	Other	2	3	5	

**Figure 2:**
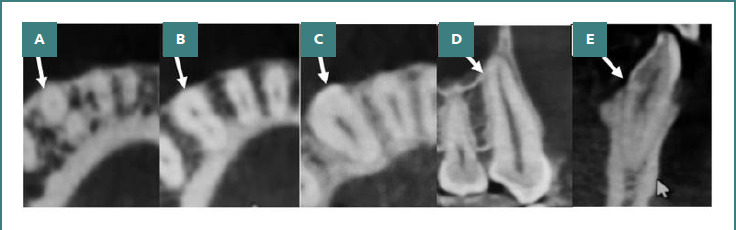
Representative cone-beam computed tomography (CBCT) images of permanent canine teeth. A, Axial section at the apical third of a mandibular canine exhibiting a bifurcated root anatomy with 2 independent root canals. B, Axial section at the middle third of a mandibular canine demonstrating a figure-of-eight canal configuration, suggestive of an isthmus or impending canal divergence. C, Axial section at the middle third of a maxillary canine displaying a characteristic oval-shaped canal morphology. D, Sagittal view of a maxillary canine illustrating a Vertucci type I configuration, characterized by a single canal extending from the pulp chamber to the apex (1-1). E, Sagittal view of a mandibular canine illustrating a Vertucci type III configuration, defined by a single canal that bifurcates within the root body before merging to exit through a solitary apical foramen (1-2-1).

The multivariable logistic regression analysis revealed that the dental arch was a significant independent predictor of canal complexity (*P* = 0.005; Table 4). Mandibular canines had significantly higher odds of exhibiting complex internal morphology compared to maxillary canines (aOR = 5.42; 95% CI, 1.68–17.45). These statistical findings are qualitatively supported by representative CBCT imaging (Figure 2A-F). Specifically, while maxillary canines predominantly exhibited simple Vertucci Class I configurations (Figure 2D), mandibular specimens frequently demonstrated more complex variations, including figure-of-eight canal patterns (Figure 2B) and Vertucci Class III (1-2-1) configurations (Figure 2F).

**Table 4:** Multivariable logistic regression analysis of factors associated with morphological variations/canal complexity in permanent canines

Variable	Adjusted Odds Ratio (aOR)	95% CI	*P* value
Arch (Mandibular vs. Maxillary)	5.42	1.68-17.45	0.005
Gender (Female vs. Male)	1.52	0.58-3.98	0.398

## Discussion

This study provides a comprehensive analysis of the root canal morphology of permanent maxillary and mandibular canines within a Saudi subpopulation in the Al-Baha region, utilizing CBCT as the primary imaging modality. A total of 120 permanent canines were examined, and the findings reveal several clinically significant morphological characteristics that are consistent with, yet distinct from, those reported in the literature.

Regarding root number, 99.16% of the examined canines were single-rooted, with only one mandibular canine presenting with two roots. This finding is largely consistent with the existing literature, which generally reports canines as predominantly single-rooted teeth. Vertucci, in his landmark classification study, established that canines are among the most consistently single-rooted teeth in the permanent dentition [6]. Similarly, Martins *et al*. reported a near-universal prevalence of single-rooted canines in a Caucasian population using CBCT analysis [3]. The systematic review by Asiri *et al*., which focused specifically on the Saudi population, further corroborated these findings, reporting that the vast majority of canines in Saudi subpopulations are single-rooted, regardless of regional variation [11].

With respect to canal configuration, our results demonstrated that mandibular canines exhibited significantly greater morphological variation than their maxillary counterparts (*P* = 0.016). Maxillary canines were predominantly classified as Vertucci Class I (93.3%). In contrast, mandibular canines showed a considerably lower prevalence of Class I configuration (71.7%), with a higher representation of Classes II, III, and V. These findings agree with those of Soleymani *et al*., who reported that mandibular canines in an Iranian population displayed a higher frequency of complex canal configurations, including Vertucci Class III, compared to maxillary canines [4]. Similarly, Basha reported analogous trends in an Egyptian subpopulation, in which mandibular canines exhibited greater internal complexity than maxillary canines [5]. The systematic review by Asiri *et al*. also highlighted the relatively higher prevalence of multiple canal configurations in mandibular canines across various Saudi subpopulations, further substantiating our findings [11]. The clinical implication of this observation is significant, as the presence of multiple canals in mandibular canines may increase the risk of missed canals during endodontic treatment, potentially compromising treatment outcomes. As emphasized by Baruwa *et al*., missed canals are a primary contributor to the prevalence of periapical lesions in endodontically treated teeth, underscoring the importance of thorough preoperative assessment using advanced imaging modalities such as CBCT [2] and potentially requiring the use of dental operating microscopes or specialized scouting files to avoid missed anatomy.

The overall prevalence of the Vertucci Class III configuration in this study was 8.5% across all canines examined, with a notably higher prevalence in mandibular canines (15.0%) than in maxillary canines (5.0%). This finding is particularly relevant from a clinical standpoint, as Class III configurations involve an initial split followed by a rejoining of canals, which can be easily overlooked during conventional radiographic assessment. Gulabivala and Ng emphasized that a thorough understanding of root canal anatomy and its variations is fundamental to achieving successful endodontic outcomes, particularly in cases involving complex canal configurations that deviate from the standard single-canal pattern [1].

Regarding gender differences, our analysis revealed no statistically significant difference in root canal complexity between male and female subjects (*P* = 0.473), with men exhibiting 14.5% complexity and women 20.7%. While women demonstrated a slightly higher percentage of complex morphology, this difference did not reach statistical significance. By employing a multivariate approach, this study controlled for gender as a potential confounder. The results indicate that the increased complexity observed in this Al-Baha subpopulation is primarily driven by tooth type (arch) rather than patient sex. Specifically, the high prevalence of oval and flattened canals in the middle third of mandibular canines (as shown in Table 3) strongly correlates with the higher Vertucci complexity. The ‘eight-shape’ configuration, found exclusively in mandibular middle thirds (*n* = 5), suggests a transition toward canal bifurcation that is absent in maxillary canines. These findings are consistent with those reported by Sert and Bayirli, who similarly found no significant gender-related differences in canal configurations among permanent canines in a Turkish population [12]. However, it is noteworthy that female subjects in our study may exhibit slightly more complex canal morphology, a trend that warrants further investigation in larger, more diverse sample populations.

The analysis of cross-sectional canal shapes at the cervical, middle, and apical thirds revealed several important findings. At the cervical third, oval-shaped canals were predominant in both maxillary (85.0%) and mandibular (90.0%) canines, with no statistically significant difference between the two arches (*P* = 0.678). This finding aligns with the established understanding that canines typically present with an oval cross-section at the cervical level due to the buccolingual width of the root. At the middle third, however, a statistically significant difference was observed between maxillary and mandibular canines (*P* = 0.0054), with maxillary canines predominantly exhibiting circular canals (86.7%) compared to mandibular canines (40.0%), which showed a higher prevalence of oval, flattened, and eight-shaped canals. This marked difference in canal shape at the middle third between maxillary and mandibular canines has important clinical implications for instrumentation and irrigation strategies. The standardized classification system utilized in this study, as described by Bueno *et al*., provided a detailed and reproducible framework for evaluating canal shapes across multiple levels, enhancing the reliability of our cross-sectional shape analysis [10]. At the apical third, no statistically significant difference was found between maxillary and mandibular canines (*P* = 0.655), with circular canals being the most prevalent shape in both arches. The most common morphological cross-sectional shape transition observed in this dataset was from oval at the cervical third to circular at both the middle and apical thirds, a pattern reported in other population-based studies using CBCT analysis [3,4].

The utilization of CBCT in this study represents a significant methodological advantage over conventional radiographic techniques, providing three-dimensional visualization of the root canal system and enabling accurate assessment of canal configurations and cross-sectional shapes at multiple levels. As advocated by Patel *et al*. and Scarfe and Farman, CBCT offers superior diagnostic accuracy to periapical radiographs for evaluating root canal morphology, particularly for identifying multiple canals, accessory canals, and complex configurations that may otherwise go undetected [8,9].

The findings of this study highlight the morphological complexity of permanent canines in a Saudi subpopulation within the Al-Baha region, with particular emphasis on the significantly higher prevalence of complex canal configurations in mandibular canines compared to maxillary canines. The absence of significant gender-related differences in canal complexity, combined with notable variation in cross-sectional canal shapes in the middle third, underscores the importance of individualized preoperative assessment with advanced imaging modalities. These findings provide a valuable clinical reference for endodontists managing patients from this specific demographic, contributing to the broader body of knowledge on root canal morphology in the Saudi population.

When comparing the findings of the current study with global data, the morphological complexity observed in the Al-Baha subpopulation aligns with patterns noted in other Middle Eastern and Asian populations, in which mandibular canines exhibit a significantly higher prevalence of multiple canals than maxillary canines (Table 5) [13-15]. While maxillary canines remain anatomically conservative across most ethnicities, the 5.42-fold increase in odds for mandibular complexity in our study underscores the clinical importance of population-specific anatomical knowledge.

**Table 5:** Comparative prevalence of Type I root canal configuration in permanent maxillary and mandibular canines across different geographical regions

Study	Region	Maxillary (Type I)	Mandibular (Type I)
Current Study (2026)	Al-Baha, Saudi	93.3 %	71.3 %
Somalinga Amardeep *et al*. (2014)[13]	India	81.6%	79.6%
Chen *et al*. (2023)[14]	China	99.2%	93.1%
Magat *et al*. (2024)[15]	Turkey	96%	92.1%

Despite the clinical relevance of these findings, several limitations must be acknowledged. First, the sample size of 120 teeth from a single center in the Al-Baha region may limit the generalizability of the results to the entire Saudi Arabian population. While this sample size is consistent with similar morphological investigations, a larger, multicenter study would provide higher statistical power to detect subtle differences in rare canal configurations. Second, the 300 μm voxel size, while sufficient for general morphological assessment, may have limited the detection of very fine accessory canals compared to high-resolution scans.

## Conclusion

This CBCT-based investigation of root canal morphology in permanent canines of a Saudi subpopulation in the Al-Baha region demonstrates that while nearly all canines were single-rooted, mandibular canines exhibited significantly greater internal morphological variation compared to maxillary canines, as evidenced by a higher prevalence of Vertucci Class II, III, and V configurations (*P* = 0.016). No statistically significant gender-related differences in canal complexity were identified. The cross-sectional shape analysis revealed a statistically significant difference at the middle third between maxillary and mandibular canines, with the most common morphological transition being oval at the cervical third to circular at the middle and apical thirds. These findings collectively emphasize the necessity of thorough preoperative CBCT evaluation and heightened clinical awareness of potential canal complexities, particularly in mandibular canines, to ensure complete canal debridement and optimize endodontic treatment outcomes in this population.

## Data Availability

The data presented in this study are available from the corresponding author upon request.

## References

[1] Gulabivala K, Ng YL (2023). Factors that affect the outcomes of root canal treatment and retreatment-A reframing of the principles. Int Endod J.

[2] Baruwa AO, Martins JNR, Meirinhos J, Pereira B, Gouveia J, Quaresma SA (2020). The influence of missed canals on the prevalence of periapical lesions in endodontically treated teeth: a cross-sectional study. J Endod.

[3] Martins JN, Marques D, Mata A, Caramês J (2017). Root and root canal morphology of the permanent dentition in a Caucasian population: a cone-beam computed tomography study. Int Endod J.

[4] Soleymani A, Namaryan N, Moudi E, Gholinia A (2017). Root canal morphology of mandibular canine in an Iranian population: a CBCT assessment. Iran Endod J.

[5] Basha S (2018). Evaluation of root canal configuration of permanent mandibular anterior teeth in Egyptian subpopulation: a cone beam computed tomography study. Egypt Dent J.

[6] Vertucci FJ (1984). Root canal anatomy of the human permanent teeth. Oral Surg Oral Med Oral Pathol.

[7] Tomson PL, Adams N, Kavanagh D, Virdee SS (2025). Non-surgical endodontics: contemporary biomechanical preparation of the root canal system. Br Dent J.

[8] Patel S, Durack C, Abella F, Shemesh H, Roig M, Lemberg K (2015). Cone beam computed tomography in endodontics–a review. Int Endod J.

[9] Scarfe WC, Farman AG (2008). What is cone-beam CT and how does it work?. Dent Clin North Am.

[10] Bueno MR, Estrela C, Azevedo BC, Junqueira JL (2020). Root canal shape of human permanent teeth determined by new cone-beam computed tomographic software. J Endod.

[11] Asiri AA, AlQahtani KW, Tarrosh MY, Shaiban AS, Al Shawkani HA, Alaajam WH (2022). Root morphology and canal configuration of permanent canines among Saudi population: systematic review and comparison with worldwide studies. Int J Gen Med.

[12] Sert S, Bayirli GS (2004). Evaluation of the root canal configurations of the mandibular and maxillary permanent teeth by gender in the Turkish population. J Endod.

[13] Somalinga Amardeep N, Raghu S, Natanasabapathy V (2014). Root canal morphology of permanent maxillary and mandibular canines in Indian population using cone beam computed tomography. Anat Res Int.

[14] Chen Y, Dai Y, Yan Z, You Y, Wu B, Lu B (2023). Morphological analysis of anterior permanent dentition in a Chinese population using cone-beam computed tomography. Head Face Med.

[15] Magat G, Uzun S (2024). Evaluation of root and root canal morphology of mandibular and maxillary canine teeth in Turkish subpopulation by cone beam computed tomography with using two classification systems. BMC Oral Health.

